# Supplementary material for: The role of endoplasmic reticulum in in vivo cancer FDG kinetics
Source: PLoS One. 2021 Jun 1;16(6):e0252422. doi: 10.1371/journal.pone.0252422 (PMC8168898; doi:10.1371/journal.pone.0252422)
Supplement: S1 Appendix — (PDF) [file pone.0252422.s001.pdf]

S1 Appendix. Robustness of the proposed reg-GN algorithm to the value of the volume fraction occupied by ER with respect to cytosol.

$\frac{V_{er}}{V_{cyt}}$	v_r	k_1	k_2	k_3	k_5	k_6
0.09	0.08	0.29 ± 0.007	0.26 ± 0.13	0.48 ± 0.25	0.47 ± 0.28	0.10 ± 0.21
0.13	0.11	0.30 ± 0.004	0.26 ± 0.10	0.47 ± 0.19	0.56 ± 0.25	0.04 ± 0.04
0.17	0.15	0.31 ± 0.003	0.30 ± 0.08	0.41 ± 0.15	0.54 ± 0.25	0.04 ± 0.05
0.21	0.18	0.31 ± 0.002	0.34 ± 0.06	0.36 ± 0.11	0.52 ± 0.22	0.03 ± 0.04
0.26	0.20	0.32 ± 0.003	0.37 ± 0.06	0.28 ± 0.11	0.48 ± 0.28	0.03 ± 0.06

We processed the FDG-PET data for mouse m1 by using the BCM with five different values of the ratio $\frac{V_{\mathrm{er}}}{V_{\mathrm{cyt}}}$. The considered values, together with the corresponding v_r are shown in the first two columns of the table. For each one of the five values, we perform fifty runs of the reg-GN algorithm by varying the initial values of the kinetic parameters, randomly selected as described in the subsection *Sensitivity analysis* of the *Results* section. In each row of the table we reported mean and standard deviation over the fifty runs of the resulting estimated values of the kinetic parameters.

We observe that the actual values of $\frac{V_{\mathrm{er}}}{V_{\mathrm{cyt}}}$ only slightly affect the final estimates of the kinetic parameters. Indeed, when $\frac{V_{\mathrm{er}}}{V_{\mathrm{cyt}}}$ is close to the reference value 0.17, the average estimates of al the parameters are close to each other. On the other hand, a too small value of $\frac{V_{\mathrm{er}}}{V_{\mathrm{cyt}}}$ may cause and higher uncertainty when estimating some of the parameters. Notice for example the higher value of the standard deviation of k_6 when $\frac{V_{\mathrm{er}}}{V_{\mathrm{cyt}}} = 0.09$.
