# Supplementary material for: The role of endoplasmic reticulum in in vivo cancer FDG kinetics
Source: PLoS One. 2021 Jun 1;16(6):e0252422. doi: 10.1371/journal.pone.0252422 (PMC8168898; doi:10.1371/journal.pone.0252422)
Supplement: S2 Appendix — (PDF) [file pone.0252422.s002.pdf]

S2 Appendix. Proof of the identifiability of BCM.

Henceforth, $\mathbb{R}_+$ shall denote the set of positive real number and $\tilde{f}(s)$, $s \in \mathbb{R}_+$, shall denote the Laplace transform of the function $f(t)$.

Assuming that suitable regularity conditions are satisfied, the Laplace transform of both sides of equation (1) and (16) leads to, respectively,

$$(s\,\boldsymbol{I} - \boldsymbol{M})\,\tilde{\boldsymbol{C}}(s) = k_1\,\boldsymbol{e}\,\tilde{C}_i(s)\,, \tag{S2.1}$$

and

$$\tilde{\mathcal{C}}_T(s) - V_b\,\tilde{C}_i(s) = \boldsymbol{\alpha}\,\tilde{\boldsymbol{C}}(s)\,, \tag{S2.2}$$

where $s > 0$, and $\boldsymbol{I}$ is the identity matrix of order 3.

Let $D_{\boldsymbol{k}}(s) := \det(s\,\boldsymbol{I} - \boldsymbol{M})$. Explicit calculation shows that

$$D_{\boldsymbol{k}}(s) := s^3 + (k_2 + k_3 + k_5 + k_6)\,s^2 + [(k_2 + k_3)\,(k_5 + k_6) + k_5\,k_6]\,s + k_2\,k_5\,k_6\ . \tag{S2.3}$$

We observe that $D_{\boldsymbol{k}}(s) > 0$ for every $s \geq 0$. Therefore, for all $s \geq 0$, the linear system (S2.1) may be solved with respect to $\tilde{\boldsymbol{C}}$ yielding

$$\tilde{\boldsymbol{C}} = k_1\,\tilde{C}_i\,(s\,\boldsymbol{I} - \boldsymbol{M})^{-1}\boldsymbol{e}. \tag{S2.4}$$

Substitution of (S2.4) into (S2.2) leads to

$$\frac{\tilde{\mathcal{C}}_T(s) - V_b\,\tilde{C}_i(s)}{\tilde{C}_i(s)} = k_1\,\frac{Q_{\boldsymbol{k}}(s)}{D_{\boldsymbol{k}}(s)} \tag{S2.5}$$

where

$$Q_{\boldsymbol{k}}(s) := \alpha_1\,s^2 + [\alpha_1\,(k_5 + k_6) + \alpha_2\,k_3]\,s + \alpha_1\,k_5\,k_6 + \alpha_1\,k_3\,k_6 + \alpha_3\,k_3\,k_5\,. \tag{S2.6}$$

Denote by $\bar{s}_{1,2}(\boldsymbol{\alpha}, \boldsymbol{k})$ the roots of the second degree polynomial $Q_{\boldsymbol{k}}$. The two equations $D_{\boldsymbol{k}}(\bar{s}_1) = 0$ and $D_{\boldsymbol{k}}(\bar{s}_2) = 0$ define a subset M_r of zero measure, contained in the space $\mathbb{R}^5_+$ of the vector parameters $\boldsymbol{k}$. Henceforth, we consider vectors $\boldsymbol{k} \in \mathbb{R}^5_+ \setminus M_r$; for these vector the polynomials $Q_{\boldsymbol{k}}(s)$ and $D_{\boldsymbol{k}}(s)$ are coprime, i.e., that they do not have any common roots.

Let $\boldsymbol{h} = (h_1, h_2, h_3, h_5, h_6)$ be a vector of kinetic parameters that produce the same measurement $\tilde{\mathcal{C}}_T(s)$. Then the left–hand side of equation (S2.5) is equal for both $\boldsymbol{h}$ and $\boldsymbol{k}$ and thus

$$k_1\,\frac{Q_{\boldsymbol{k}}(s)}{D_{\boldsymbol{k}}(s)} = h_1\,\frac{Q_{\boldsymbol{h}}(s)}{D_{\boldsymbol{h}}(s)}\,, \tag{S2.7}$$

where $D_{\boldsymbol{h}}$ and $Q_{\boldsymbol{h}}$ are defined by substitution of $\boldsymbol{k}$ with $\boldsymbol{h}$ in (S2.3) and (S2.6).

It is found from the necessary condition (S2.7) that the equation $h_1 D_{\boldsymbol{k}}(s) Q_{\boldsymbol{h}}(s) = k_1 D_{\boldsymbol{h}}(s) Q_{\boldsymbol{k}}(s)$ holds. Comparison of the leading order terms shows that $h_1 = k_1$. As an immediate consequence, equation (S2.5) leads to the necessary condition

$$\frac{Q_{\boldsymbol{h}}(s)}{D_{\boldsymbol{h}}(s)} = \frac{Q_{\boldsymbol{k}}(s)}{D_{\boldsymbol{k}}(s)}\ . \tag{S2.8}$$

The two ratios have the same zeros. Because of coprimality, this holds also for the polynomials $Q_{\boldsymbol{h}}(s)$ and $Q_{\boldsymbol{k}}(s)$, whence it follows straightforwardly that

$$Q_{\boldsymbol{h}}(s) = Q_{\boldsymbol{k}}(s), \qquad D_{\boldsymbol{h}}(s) = D_{\boldsymbol{k}}(s). \tag{S2.9}$$

Eqs. (S2.9) give rise to the overdetermined polynomial system

$$h_2 + h_3 + h_5 + h_6 = k_2 + k_3 + k_5 + k_6 \tag{S2.10}$$

$$(h_2 + h_3)\,(h_5 + h_6) + h_5\,ha_6 = (k_2 + k_3)\,(k_5 + k_6) + k_5\,k_6 \tag{S2.11}$$

$$h_2\,h_5\,h_6 = k_2\,k_5\,k_6 \tag{S2.12}$$

$$\alpha_2\,h_3 + \alpha_1\,(h_5 + h_6) = \alpha_2\,k_3 + \alpha_1\,(k_5 + k_6) \tag{S2.13}$$

$$\alpha_1\,h_5\,h_6 + \alpha_2\,h_3\,h_6 + \alpha_3\,h_3\,h_5 = \alpha_1\,k_5\,k_6 + \alpha_2\,k_3\,k_6 + \alpha_3\,k_3\,k_5 \tag{S2.14}$$

of five equations for the four unknowns h_2, h_3, h_5, h_6.

Clearly, $\boldsymbol{h} = \boldsymbol{k}$ solves the system (S2.10)-(S2.14). Long and cumbersome calculations show that solutions with $\boldsymbol{h} \neq \boldsymbol{k}$ are allowed only if $\boldsymbol{k}$ belongs to $M_0 \subset \mathbb{R}^5_+$, where M_0 is a suitably defined set of zero measure.

We conclude that the proof that $\boldsymbol{h} = \boldsymbol{k}$ holds in $\mathbb{R}^5_+ \setminus (M_r \cup M_0)$, that is, almost everywhere in $\mathbb{R}^5_+$. This suffices to state that the BCM is structurally globally (or uniquely) identifiable [1].

References

[1] Chis OT, Banga JR, Balsa-Canto E. Structural identifiability of systems biology models: a critical comparison of methods. PloS one. 2011;6(11):e27755.
