# Supplementary material for: The role of endoplasmic reticulum in in vivo cancer FDG kinetics
Source: PLoS One. 2021 Jun 1;16(6):e0252422. doi: 10.1371/journal.pone.0252422 (PMC8168898; doi:10.1371/journal.pone.0252422)
Supplement: Attachment — Submitted filename: Response_to_reviewer.pdf [file pone.0252422.s003.pdf]

EDITOR

Dear Authors,

I, as Editor, very much respect the route where you embarked. As you see the reviewers were substantially criticizing your work and rightly so, especially in not seeing the results of real-world measurements and not finding well substantiated references for estimations. However I think your work is highly important for both the biochemistry, the cell biology and the broader Nuclear Medicine/Molecular Imaging community and your basic concept is worth pursuing. So I propose a very substantial major revision instead of rejecting the manuscipt. Please especially pay attention to deal with the statements regarding the BCM model vs. the SCM model and the data available with 14C-glucose, 3H-glucose from 30 years ago, or your own experimental measurements in vitro, not just estimations.

I do look forward to receiving your substantially rewritten major-revision.

Dear Editor,

We greatly appreciate your interest in our work and both reviewers' effort in reading our manuscript and providing constructive suggestions.
Following their comments we substantially revised our manuscript. In particular, we have more carefully interpreted the obtained results in light of our own in vitro experiment as well as of previous studies employing a Sokoloff's compartment model similar to the one used here.
Additionally, we now also provide all code and experimental data required to replicate our study. Specifically, all relevant material. including the input function C_i and the total concentration C_T from 6 murine measurements, has been deposited in the publicly available git-hub repository
https://github.com/theMIDAgroup/BCM_CompartmentalAnalysis.git.
Please, find below a point-by-point answer to the reviewer's concerns. In the following, the reviewers' comments are reported in bold, the authors' responses are given in plain text, while italic text represents extracts from the new version of the manuscript.

Reviewer #1:

The main difficulty with the problem in the article is that on a macroscopic measurement like PET for concentration, it is not possible to provide data for phosphorilated, non phosphorilated, cytosol and ER fractions. Therefore only C_T can be used. This precludes the claim of the authors that the BCM model can be proven to be better fit than the SCM model.
The referee is right: the way we concluded the Abstract and the Introduction in the submitted version of the paper was clearly too apodictic and did not exactly correspond to what our results really can explain. In the amended version of the manuscript we have notably rephrased these parts, trying to better keep the point about the connection between our biochemically-driven assumption and what the data

and results described in the text really state.

Specifically, we

- modified the last part of the Abstract that now reads as follows.
  This analysis shows that the proposed three-compartment model equals the performance of a standard Sokoloff's two-compartment system in fitting the data. However, it provides estimates of some of the parameters, such as the phosporilation rate of FDG, more consistent with prior biochemical information. These results are made more solid from a computational viewpoint by proving the identifiability and by performing a sensitivity analysis of the proposed compartment model
- modified the Introduction from line 43 to line 56, that now read as follows.
  The main objective of the present paper is to discuss the reliability of the proposed three-compartment model for the analysis of FDG kinetics in in vivo tissues. To this end we consider FDG Positron Emission Tomography (FDG-PET) data of murine models inoculated with specific murine cancer cells. Precisely, we have processed six datasets provided by a PET scanner for small animals in the case of six murine models of CT26 colon cancer. The compartment model used for this analysis is the analog of the one utilized in [12] for describing the FDG kinetics in the case of in vitro cultured data, and is designed according to the biochemically-driven assumption that most FDG is dephosphorylated in ER. As a validation, the results provided by the proposed approach are compared with those obtained through a standard Sokoloff's two-compartment model, and with the results from the in vitro experiment where direct verification is possible. Further, the reliability of the results is corroborated by identifiability considerations based on a formal and numerical analysis of the compartmental equations.

When determining the volume fraction of ER to cytoplasma, v_r, in the article it must be referenced, or reasoned of why the value given v_r = 0.14. The text says estimated as 0.14, however the specific value will determine the exact C_er and C_c concentrations heavily, thus this estimate has to be very solid. Was it optimized together with the k_i parameters? Or taken as typical values from cell biology of these cancer cell lines? Or used as a hand-tuned hyperparameter that gives the best fit?

We agree with the referee and in order to amend the new version of the paper along these suggestions we followed two approaches.
First, we better justified the choice $v_r = 0.14$ within the main text at page 5 line 119-125. In details we rewrote v_r in terms of the ratio $V_{\mathrm{er}}/V_{\mathrm{cyt}}$ whose value has been set equal to 0.17 according to the following bibliographic sources that we added to the new version of the bibliography:

19. Alberts B, Johnson A, Lewis J, Raff M, Roberts K, Walter P. Molecular biology of the cell, 5th edn. Garland Science, New York; 2008.
20. Milo R, Phillips R. Cell biology by the numbers. Garland Science, New York; 2015.

Second (and, probably, more significantly), we tested numerically the robustness of the proposed inverse scheme with respect to the value of $V_{\rm er}/V_{\rm cyt}$. More in detail, we considered 5 different values of $V_{\rm er}/V_{\rm cyt}$, namely we set $V_{\rm er}/V_{\rm cyt} = 0.17\alpha$ with $\alpha \in [0.5, 0.75, 1, 1.25, 1.5]$. For each one of these values we solved the inverse problem for mouse m_1 so as to estimate the values for the kinetic constants k_1, k_2, k_3, k_5, k_6.
The outcome of this numerical experiment is illustrated in S1 Appendix. Our results show that the solution of the compartmental inverse problem weakly depends on the value of v_r.

"A straightforward computational analysis showed.....very few and totally unrealistic". You have to provide numerical probabilities for "very few", and numbers for the physiological ranges that these exceed thus becoming "unrealistic".
The reason why we used these blurred expressions is because we tried to avoid including a formal mathematical result we could prove. This theorem shows that the identifiability holds true for all 5-tuple in $\mathbb{R}^5_+$, except a set of values of the kinetic coefficients with zero measure. The amended version of the subsession *Identifiability issues* now contains a description of this result, whose details are now given as supplementary material, see S2 Appendix.
Additionally the following reference has been added to support the definition of identifiability we employed.

31. Chis OT, Banga JR, Balsa-Canto E. Structural identifiability of systems biology models: a critical comparison of methods. PloS one. 2011;6(11):e27755

k5 and k6 are somewhat underdetermined parameters, and also depend on the volume fraction of ER to total cell. So these parameters having 2 undetermined values will of course give a huge plane of solutions yielding the same C_T. The initial condition and soft bound method is okay to work with, though, but see the paragraph about cell biological constraints of my review on relevant ranges. You also have to provide numerical values for the k6 impact and provide numerical estimate for stating it is negligible.

We thank the reviewer for pointing this out. In fact, our sensitivity analysis shows that k_5 and k_6 are the parameters characterized by the lowest sensitivity. To cope with this issue we introduce a constraint on the initial values of k_5 and k_6 provided to the proposed reg-GN iterative scheme.
More in detail, we set the initial value of k_6 equal to 0 according to various bibliographic sources that reported a value of the rate of dephosphorylation of FDG6P of the order of 10^-2 (1/min) or lower. However, it is worth noticing that this does not imply neglecting k_6. Its actual value is estimated by the reg-GN algorithm and is in fact greater than zero, see e.g. Table 2.
Instead, we are not aware of any bibliographic source on compartmental analysis reporting a range of biologically feasible values of k_5. In fact, to the best of our

knowledge, no previous work presented a compartmental analysis on the role of the ER in the accumulation of the phosphorylated tracer in tissues. For this reason, to initialize k_5 we rely on a constraint solely based on our sensitivity analysis. Moreover, as shown in S1 Appendix, when we implement such constraints, the actual value of the volume fraction of ER to cytosol does not significantly impact the final estimated values of the kinetic parameters.
However, we reckon that this was unclear and not well motivated in the previous version of the manuscript. For this reason we thoroughly revise the subsection Sensitivity analysis, pag 8, lines 215-228, that now reads as follows:

To account for these results we introduced a constraint on the values used to initialize k_5 and k_6 within the reg-GN algorithm. In detail, we initialized k_5 according to the following simple heuristic procedure. First we processed the same experimental data by using the standard SCM, which provides a first estimate of the four kinetic parameters k_1, k_2, k_3, k_4; then, in BCM, we set the initial value of k_5 equal to a random positive number lower than $\sqrt{(k_1)^2 + (k_2)^2 + (k_3)^2 + (k_4)^2}$, where the values of these four parameters were estimated by SCM. This initialization condition implements an energetic constraint on k_5, which prevents it from reaching ranges of values where the sensitivity of C_T becomes too small. Instead, the initial value of k_6 was set equal to 0 in order to promote small estimates of such a parameter. This choice was supported by results shown in previous works where the dephosphorylation rate of FDG6P was assumed to be zero [4, 33] or estimated of the order up to 10^-2 (1/min) [34, 35].
The initial values of k_1, k_2, k_3 of BCM and of the four parameters of SCM, were randomly drawn in (0, 1).

The following references have been added to justify our choice on the initial value of k_6:

33. Ishibashi K, Wagatsuma K, Ishiwata K, Ishii K. Alteration of the regional cerebral glucose metabolism in healthy subjects by glucose loading. Hum Brain Mapp. 2016;37(8):2823–2832.

34. Reivich M, Alavi A, Wolf A, Fowler J, Russell J, Arnett C, et al. Glucose metabolic rate kinetic model parameter determination in humans: the lumped constants and rate constants for [18F]fluorodeoxyglucose and [11C]deoxyglucose.415J Cereb Blood Flow Metab. 1985;5(2):179–192.

35. Dimitrakopoulou-Strauss A, Pan L, Sachpekidis C. Kinetic modeling and parametric imaging with dynamic PET for oncological applications: General considerations, current clinical applications, and future perspectives. Eur J Nucl Med Mol Imaging. 2020; p. 1–19

On a more general note, Figure 3 should be redesigned and much better reflect multiple parameter dimension sweeps. At the moment this is a very small area of the 6D parameter space that is shown. These graphs have to be convincing that the results are robust to the qualitative parameter transitions (e.g. 3(c) inversion at time = 10 min).

We thank the reviewer to point this out. We replaced panel (a)-(e) with a 3D version where a larger number of parameters are considered. Additionally, we revised the first part of the section Sensitivity analysis pag 7 lines 192-202. In the amended version, we better justify the ranges of tested values of the kinetic parameters, and we more clearly describe the results obtained.
We also point out that our inversion approach reg-GN requires in input the whole curve C_T. For this reason, it results to be weakly affected by sensitivity issues that concern only a single time point, as for example time=10min in panel (c).

I would also like to see some of the constraints and results presented in this macroscopic in vivo article explained numerically from the in vitro study, where accumulation of cellular compartments is possible. Even if FDG is not available, Glucose and Gl6H typical cellphysiological numerical values should guide both the constraints of the model, and the results should be compared to. This is essential to show that the BCM model indeed captures cellphysiologically relevant fractional compartmental kinetics, rather than arbitrarily fitting a high order polynomial to a nonlinear C_T graph. In fact the more biological evidence you can marshall behind specific values of the chosen parameters and the results, the fitted and found parameters, the less is required to evidence that the method is a relevant better model than the two compartment model.

We thank the reviewer for raising up this important point. As already pointed out previously in this letter, in the amended version of the manuscript we highlighted the bibliographic sources motivating the imposed constraints. See, in particular, our answers on the choice of the volume fraction v_r and on the constraints imposed on the initial values of k_5 and k_6.
Moreover, we substantially revised both the *Discuss* and the *Conclusion* section in order to interpret the obtained results in light of previous studies. In particular, the subsection *'Comparison between SCM and BCM'* of the submitted version has been replaced by two subsections entitled *'Kinetic parameters and comparison with previous works'* and *'Time curves of the compartment concentrations'*. In these new sections, the results obtained in terms of the reconstructed kinetic parameters and the compartment concentrations were systematically compared with those provided by the in vitro study. Additionally, as a further validation of the proposed numerical inversion technique, the results obtained with the SCM have been compared with those from previous works employing a Sokoloff-type compartment model.

The article must contain significantly detailed description of how the measured concentration data is consolidated in space to fit the model. At the moment there is nothing about it.

To account for this suggestion, in the section *Tracer kinetics* of the amended version of the manuscript we have better explained how, starting from the estimated parameter vector $\mathbf{k}$, it is possible to reconstruct the concentrations of the different compartments and then the total concentration C_T. As shown in the new Figure 4, panel (a) and (b), the obtained total concentrations correctly fit the experimental data for both BCM and SCM. We hope this answers the Reviewer's concern.

Fig 4 shows a very contradictory image of the comparison of the BCM and SCM models. First the concentration graphs should include the total concentration per tissue volume, C_T, as well as the data so that it can be seen that they fit well. Second the detailed concentration graphs should also including interstitial and blood fractions individually. Third the grand total of the intracellular concentrations in these graphs are not comparable. In fact they converge to completely different cellular total concentrations differing in a factor of 3-4. The models should not be different with cell in and out parameters, nor with the interstital and blood concentrations, and therefore not with the intracellular concentrations. I recommend a total volume check as well.

The referee is right. We thoroughly revised Fig. 4 following these suggestions. More in detail, the figure is now structured in the following 6 panels.

- In panel (a) and (b) we compare the experimental total concentration $\mathcal{C}_T$ and the curves $\hat{\mathcal{C}}_T$ reconstructed by using the BCM and the SCM, respectively. We quantify the quality of the fit by computing the relative error
$$\frac{||\mathcal{C}_T - \hat{\mathcal{C}}_T||_2}{||\mathcal{C}_T||_2}$$
for 50 different runs of the inversion scheme. In the BCM such an error remains always below 0.13 for SCM it remains below 0.17.
- In panel (c) we plot the concentrations of the compartment C_f, C_p and C_r of BCM and in panel (d) the concentrations C_f and C_p of SCM. These panels correspond to panels (a) and (b) of the previous version of the manuscript and describe the time course of the natural state variables of tracer kinetics. As observed in the text, these graphs highlight that while in SCM the accumulation of the phosphorylated tracer occurs in the cytosol, in BCM it occurs in the ER. As noticed by the reviewer, the value of C_r in panel (c) is about four times as high as the intracellular concentration in SCM. This is ultimately due to the fact the reticulum occupies a smaller ratio of the intracellular volume with respect to the cytosol and thus the same amount of tracer produces a higher concentration in the reticulum than in the cytosol. To remove this ambiguity, we added the panels (e) and (f) described below.
- In panel (e) we show the contribution to the total concentration $\mathcal{C}_T$ due to the tracer in the interstitium, the cytosol and the reticulum estimated with the BCM. Such contributions can be defined, by manipulating equation (8) and decomposing $\mathcal{C}_T$ as
$$\mathcal{C}_T = \frac{V_{\text{blood}}}{V_{\text{tot}}}C_i + \frac{V_{\text{int}}}{V_{\text{tot}}}C_f + \frac{V_{\text{cyt}}}{V_{\text{tot}}}(C_f + C_p) + \frac{V_{\text{er}}}{V_{\text{tot}}}C_r$$
The last three addends are respectively the contribution of the interstitial fluid, cytosol and reticulum.

We did not report the contribution due the blood, i.e. $\frac{V_{\mathrm{blood}}}{V_{\mathrm{tot}}}C_i$, as it is assumed as known and can be seen in figure 2 (c).
For the ease of comparison panel (f) shows the corresponding contributions estimated with the SCM.
As expected, panel (e) and (f) show that the contribution due the tracer in the interstitium is similar in the two models, BCM and SCM, and the grand total of the intracellular concentrations, i.e. the cytosol for SCM and the sum of cytosol and reticulum for BCM, tends to the same values.

Reviewer #2

The main problem in this article is that the measurement like PET for concentration, it is not at all possible to provide data for phosphorylated, non-phosphorylated, cytosol and ER fractions.
The authors failed to provide significant and justified presentation of their data and content!

The Reviewer is right, one of the difficulties in the validation of our results is that *in vivo* PET measurements do not allow to measure the concentrations of the different compartments. In fact, compartmental analysis can be used to obtain indirect measurements of such concentrations. In the amended version of the manuscript we use results from previous works to systematically support the constraints we imposed on the model and to validate our analysis.
More in detail, the results obtained with the proposed three-compartment model have been compared to those of a standard Sokoloff's model applied on the same experimental dataset. Indeed, for Sokoloff's model reference values can be found from previous studies applying similar models in the case of other kinds of carcinoma, for both mice and humans. Additionally, the results obtained with the proposed three-compartment model have been compared with those from a previous experiment *in vitro* on cultured cells where direct measurements were possible.
The results of our analysis indicated that with the proposed BCM model we are able to correctly fit the measured total concentration, while providing estimates of some of the parameters (for example the rate of phosphorylation of FDG) more consistent with biological data than those from the Sokoloff model.
Accordingly, we notably rephrased different parts of the Manuscript, trying to better keep the point about the connection between our biochemically-driven assumption and what the data and results described in the text really state.
More details can be also found in the answers to the concerns of Reviewer #1.

The Figure 3 should be reformulated in much much better understanding for multiple parameter dimension. Practically this is a very little area of the 6D parameter space that has been shown

Also Reviewer #1 rised a similar point. We refer to the associated answer that we report here:

We replaced panel (a)-(e) with a 3D version where a larger number of parameters are considered. Additionally, we revised the first part of the section Sensitivity analysis pag

7 lines 192-202. In the amended version, we better justify the ranges of tested values of the kinetic parameters, and we more clearly describe the results obtained.
We also point out that our inversion approach reg-GN requires in input the whole curve C_T. For this reason, it results to be weakly affected by sensitivity issues that concern only a single time point, as for example time=10min in panel (c).
